# Affordable Nutrient Solutions for Improved Food Security as Evidenced by Crop Trials

**DOI:** 10.1371/journal.pone.0060075

**Published:** 2013-04-02

**Authors:** Marijn van der Velde, Linda See, Liangzhi You, Juraj Balkovič, Steffen Fritz, Nikolay Khabarov, Michael Obersteiner, Stanley Wood

**Affiliations:** 1 International Institute for Applied Systems Analysis (IIASA), Ecosystem Services and Management Program, Laxenburg, Austria; 2 International Food Policy Research Institute (IFPRI), Washington D.C., United States of America; 3 College of Economics and Management, Huazhong Agricultural University, Wuhan, China; 4 Global Development Program, Bill & Melinda Gates Foundation, Seattle, Washington, United States of America; Centro de Investigación y de Estudios Avanzados del IPN, Mexico

## Abstract

The continuing depletion of nutrients from agricultural soils in Sub-Saharan African is accompanied by a lack of substantial progress in crop yield improvement. In this paper we investigate yield gaps for corn under two scenarios: a micro-dosing scenario with marginal increases in nitrogen (N) and phosphorus (P) of 10 kg ha^−1^ and a larger yet still conservative scenario with proposed N and P applications of 80 and 20 kg ha^−1^ respectively. The yield gaps are calculated from a database of historical FAO crop fertilizer trials at 1358 locations for Sub-Saharan Africa and South America. Our approach allows connecting experimental field scale data with continental policy recommendations. Two critical findings emerged from the analysis. The first is the degree to which P limits increases in corn yields. For example, under a micro-dosing scenario, in Africa, the addition of small amounts of N alone resulted in mean yield increases of 8% while the addition of only P increased mean yields by 26%, with implications for designing better balanced fertilizer distribution schemes. The second finding was the relatively large amount of yield increase possible for a small, yet affordable amount of fertilizer application. Using African and South American fertilizer prices we show that the level of investment needed to achieve these results is considerably less than 1% of Agricultural GDP for both a micro-dosing scenario and for the scenario involving higher yet still conservative fertilizer application rates. In the latter scenario realistic mean yield increases ranged between 28 to 85% in South America and 71 to 190% in Africa (mean plus one standard deviation). External investment in this low technology solution has the potential to kick start development and could complement other interventions such as better crop varieties and improved economic instruments to support farmers.

## Introduction

*Farming looks mighty easy when your plow is a pencil and you’re a thousand miles from the corn field.* –Dwight D. Eisenhower, 1956.

**Figure 1:**
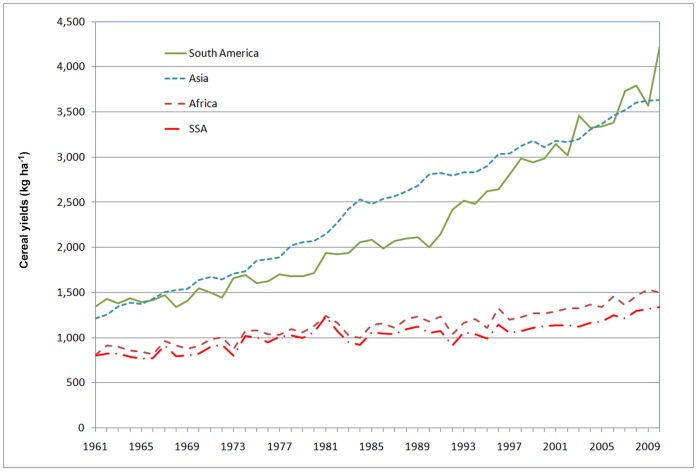
Cereal yield trends since 1960 in Africa, Sub-Sahara Africa (SSA), South America and Asia.

The increases in global population and food demand clearly indicate that current growth in agricultural productivity is not sufficient to sustain the 9 billion people that will inhabit the Earth by 2050 [1]. Feeding the world is a multifaceted and complex challenge and a number of solutions have been offered, where closing the yield gap is one of the most frequently cited recommendations [2]; [3]; [4]; [5]; [6]. The FAO [1] suggests that 70% of the required increase in crop production in developing countries should be realized through boosting the productivity of fields already under cultivation. Without this intensification, the inevitable cropland expansion will lead to deforestation, accelerate land degradation and threaten natural habitats and biodiversity [7]. A large part of this augmentation in crop production must therefore come from soils in tropical regions which are often highly weathered, have low levels of chemical soil fertility and will need additional inputs to improve crop productivity [8]. Global fertilizer use has already increased significantly since 1960 [9] and this increase has played an important role in the Green Revolution, benefitting many developing countries in South America and Asia. Yet substantial progress is still lacking in Africa [10]. From 1960 to 2000, yields of staple crops such as wheat, rice and corn increased in South America by over 180% while African yields did not improve substantially (see Figure 1). These contrasting trajectories reflect disparities in infrastructure development, primary crop types grown, agricultural R&D and extension capacities, socio-economic conditions as well as environmental differences [10]; [11]; [12].

In many smallholder fields, fertilizer and manure inputs have been too low for too long [8]. Agricultural soils cultivated without adequate nutrient replenishment cannot reach their full crop production potential and are at risk of irreversible degradation [13]. In large parts of Africa, this has led to seemingly perpetual low per capita food production [8]. Without maintaining adequate soil fertility levels, crop yields cannot be sustained, increase over time, or respond to improved agricultural management practices. To improve nutrient input, smallholder farmers need actionable strategies such as micro-dosing: applications of small quantities of fertilizers. Field studies have shown that micro-dosing presents an attainable strategy for smallholder farmers in line with their financial means that can result in significant yield gains [14]. Importantly, previous higher fertilizer rate recommendations have ignored the sizeable but unlikely investment that would be required by poor and risk adverse smallholders.

Fertilizer prices in Africa are often higher relative to other developing countries. The small size of the fertilizer market, the high transportation and handling costs and the inefficient supply chain all contribute to the relatively high retail prices for fertilizers in Africa [15]. Fertilizer price also varies across regions, through different years and even among cropping seasons in the same year. In a landlocked country such as Uganda, the prices for urea in 2000 ranged from 600 shillings kg^−1^ (300 US$ ton^−1^) in the central districts to over 750 shillings kg^−1^ (375 US$ ton^−1^) in the eastern districts while prices for phosphate (Diammonium Phosphate, DAP) ranged from 560 shillings kg^−1^ (280 US$ ton^−1^) in the long rainy season to over 700 shillings kg^−1^ (350 US$ ton^−1^) in the short rainy season [16]. As a comparison, US farmers in 2000 paid from US$80 to US$120/ton for Urea, and US$140 to US$170/ton for DAP. Depending on the locations and seasons, the NPK (Nitrogen-Phosphorus-Potassium) compound fertilizer ranged from 700 shillings kg^−1^ (350 US$ ton^−1^) to over 1000 shillings kg^−1^ (500 US$ ton^−1^) for 1∶1:2 NPK, the most common compound fertilizer in Uganda. The transportation cost in Uganda is over one third of the total fertilizer cost while it is about a fifth in Tanzania [17] due to the major sea port of Dar Es Salaam. Moreover, there are global pressures that lead to price volatility in fertilizer prices. For example, there was a fourfold increase in the price of urea from 2000 to 2008, reaching over 500 US$ ton^−1^, falling to around 200 US$ ton^−1^ in 2009 and which is currently at around 400 US$ ton^−1^.

To formulate more realistic sustainable intensification pathways, we need better estimates of smallholder yield gaps in tropical countries and to then align these with local fertilizer prices and associated investment costs. There are a number of yield gap approaches that estimate different types of attainable yield potentials across varying spatial and temporal scales [18]. Many global assessments of yield gaps use crop models or data that currently lack sufficiently detailed spatial information on soil characteristics, crop management practices and crop responses to fertilizers [19], [20]. Mueller et al. [19] found that large crop production increases are possible, but will require considerable changes in nutrient and water management. Crop trials represent a valuable source of information for yield gap analysis and could be analyzed more comprehensively for this purpose, yet are rarely collected systematically. Furthermore, nutrient specific analysis of the relationships between fertilizers and crop yields has been limited, especially in the tropical and subtropical regions where crop yields are relatively low and must increase the most to meet growing demands [21].

**Figure 2:**
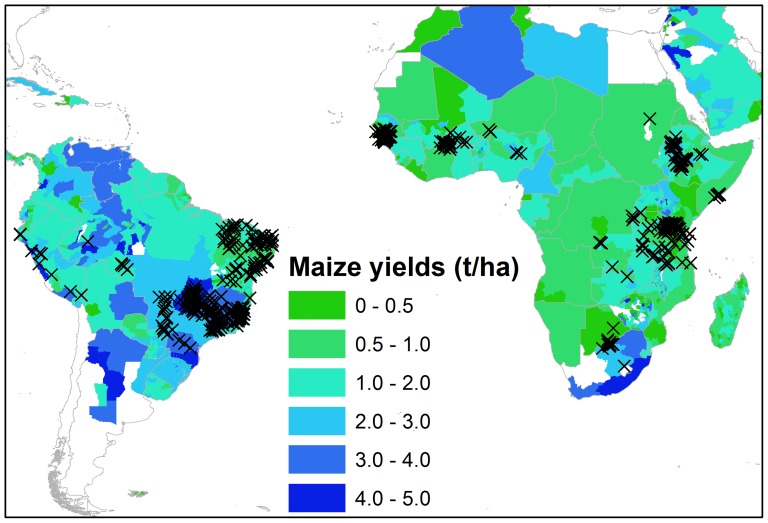
Crosses indicate locations of 1358 historic FAO corn field trials with at least five N and P input combinations in Africa and South America carried out between 1969 and 1993. Colors indicate (subnational) maize (corn) yields (ton/ha) as collected by [23].

In this paper we analyze historic data from FAO corn fertilizer trials carried out between 1969–1993 at 1358 locations in Africa and South America (Figure 2) where corn is the most commonly cultivated crop [18]. Mitscherlich-Baule crop response functions were fit to the crop trial data by optimizing the factors describing yield responses to elemental nitrogen (N) and phosphorus (P) inputs as well as an initial (residual) soil N and P. Nitrogen and Phosphorus specific fertilizer application rates from [22] were then used as inputs to the crop response functions, and the resulting yields were validated using sub-national yield statistics on corn from the International Food Policy Research Institute (IPFRI) [23] to estimate yield gaps in corn at the continental level. Only water-limited (i.e. rain fed) yield potential as opposed to irrigated yields potentials are considered here. Furthermore, we consider the importance of soil nutrient stoichiometry and the viability of micro-dosing [14], [24] in order to further the African crop productivity discourse [25], [26]. Yield increases associated with two different scenarios are considered here: 1) depicting a micro-dosing strategy and 2) a topping up to a conservative estimate of average nutrient fertilizer rates in the USA. Finally, the investment costs of scaling up these scenarios are calculated using the latest average fertilizer prices in Africa and South America and used to evaluate whether these approaches can function as part of an actionable development blueprint for Sub-Saharan Africa.

## Materials and Methods

### Crop Trials and Response Functions

Recently historic FAO crop fertilizer field trials have become publically accessible (http://www.fao.org/ag/agl/agll/nrdb/). These data were collected as part of FAO’s Fertilizer Programme [27] that ran from 1969 until 1993. The purpose behind the programme was to undertake trials to determine suitable fertilizer application rates for locally grown crops and to demonstrate to as many farmers as possible, the positive effect of fertilizer application on crop yields and farm income. The information available from the trials includes crop yields (kg ha^−1^), and application rates of the main nutrients (nitrogen (N), phosphorus (P) and potassium (K) and farmyard manure (kg ha^−1^)). Unfortunately, detailed soil or meteorological information was not recorded. Nitrogen was mostly applied as urea, phosphorus (P) was mostly applied as superphosphate and potassium as part of NPK compound fertilizers. All the applied nutrients were recalculated to elemental application rates (with P calculated from P_2_O_5_ and K from K_2_O). The application rates of farm yard manure were converted to N, P-P_2_O_5_ and K-K_2_O application rates following [28]. Data on corn yields from trials with at least five N and P input combinations were selected for this analysis. The Mitscherlich-Baule crop response function was used to analyze relations between nitrogen (N) fertilizer input, phosphorus-phosphate (P) fertilizer input and corn yields *y_mb_*:
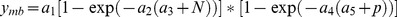
(1)


The function allows for growth that plateaus with increasing fertilizer application, and accommodates cases of both near perfect factor substitution and near zero factor substitution, and performs superior to a quadratic and von Liebig type production function [29]. The growth plateau is represented by a_1_, which was set equal to the maximum yield obtained in each field trial, while a_3_ and a_5_ represent the residual available nitrogen and phosphorus in the soil. Taking account of residual soil phosphorus is important; the cumulative cropland P surplus in certain countries in Western Europe has led to a buildup of residual soil P with expected future benefits to crop production, although lower and no effects are generally expected for Latin America and Africa [30]. The coefficients a_2_ and a_4_ describe the influence of the corresponding N or P fertilization on yield. The parameters a_2_, a_3_, a_4_, and a_5_ were obtained by minimizing the sum of squared errors for all applications in each experiment. The Nelder-Mead multidimensional unconstrained nonlinear minimization algorithm was used to minimize the objective function. Only those trials where a crop response function could be fit were used. This resulted in a total of 1358 unique experiments with at least five N and P input combinations; 752 in Africa and 606 in South America.

### Current Fertilizer Application Rates

The fertilizer dataset of [22] containing crop fertilizer rates was used to assign the current N and P inputs from chemical fertilizer (Nfer, Pfer) and manure (Nman, Pman; see Figures S1, S2, S3) at the trial locations and considered representative for corn fertilizer rates [31]. Data on corn yields collected by IFPRI [23] were used as a comparison with the yields obtained from the crop response functions (Figure S4). The average cereal area, production and yield as reported by FAOSTAT for 2008–2010 were calculated for each of the scenarios. In the area scenario a constant production was assumed and an increase in yield would reduce the requirement for cropland. In the people scenario an increase in yield would produce more on the same cropland area. We assume the average cereal calorie content to be 3000 kcal kg^−1^ and the average annual calorie need of a person to be 1 million kcal year^−1^.

### Costs

The cost of the two proposed scenarios was calculated using the Agricultural GDP in US dollars for 2009 [32]: South America (SA) $192 billion USD; Sub-Saharan Africa (SSA) $150 billion USD. Then the costs for each region were calculated by taking the total cost, dividing by the Agricultural GDP and multiplying by 100 to arrive at the percentage of Agricultural GDP that would be required to finance the scenario.

## Results

**Figure 3:**
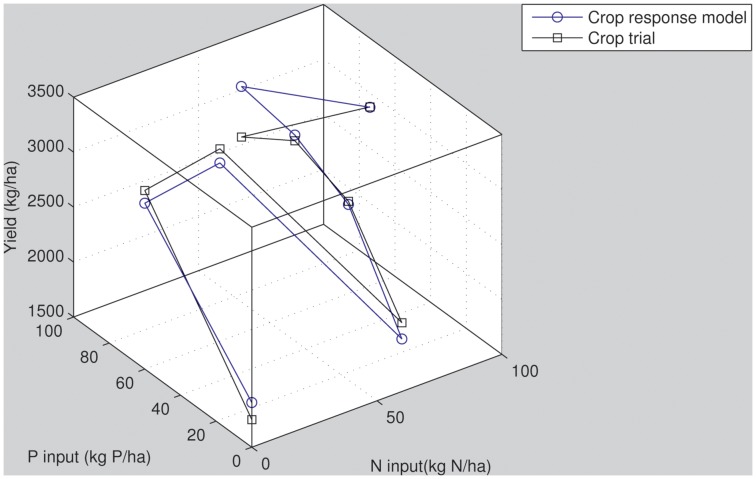
A typical example of a fitted crop response trial with experimental (blue line with circles) and modeled data (black line with squares) with eight N and P input combinations and resulting yields.

An example of crop response trial data and the corresponding modeled Mitscherlich-Baule crop response function is shown in Figure 3. The median r^2^ obtained by fitting the individual crop trials equaled 0.81; the 25^th^ percentile equaled 0.66 and the 75^th^ percentile 0.91. The resulting median, 25^th^ and 75^th^ percentiles for the a_1_, a_2_, a_3_ and a_4_ parameters obtained across all crop trials are presented in Table 1. Median values corresponded to 0.017 ton kg^−1^, 68.4 kg N ha^−1^, 0.29 ton kg^−1^, 3.18 kg P ha^−1^ for parameters a_1_, a_2_, a_3_ and a_4_ respectively. This is in correspondence with the parameter values reported by [29] and [33]. The results from the individual crop trials indicate that out of the 1358 trials, there were 1037 trials (76%) that responded stronger to added phosphorus than nitrogen. Similarly, for 82% of the trials the pool of residual soil N was larger than the accessible residual P. Clearly, these site-specific analyses indicate that overall, phosphorus is the nutrient most limiting crop yield. Nevertheless, at the same time, the range of parameter values obtained highlights the variety of crop yield responses depending on site-specific conditions.

**Table 1:** The median, 25^th^ and 75^th^ percentile values from the distributions of the Mitscherlich-Baule crop response function parameters (a_1_, a_2_, a_3_ and a_4_) fitted for the 1358 individual crop trials.

Parameter	Median	25^th^ percentile	75^th^ percentile
a1	0.017321	0.0077335	0.047077
a2	68.4297	21.6344	285.0599
a3	0.29219	0.047732	0.29219
a4	3.1803	0.99095	13.0806

**Figure 4:**
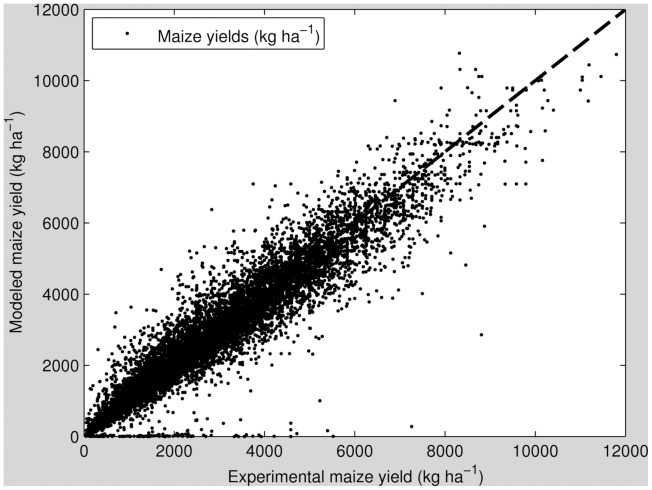
Relationship between historic FAO experimental corn field trials with at least five N and P input combinations and corn yields calculated with the Mitscherlich-Baule crop response function totaling 1358 unique nutrient-yield relations (r^2^ = 0.94).

Overall, the yields modeled using Mitscherlich-Baule crop response functions show a good relationship (*r^2^ = 0.94*, Figure 4).

### Yield Gaps and Potentials

The fertilizer dataset of Potter et al. [22] was used to assign the current N and P inputs from chemical fertilizer (Nfer, Pfer) and manure (Nman, Pman) to the crop response functions; the average of these inputs and their distribution across Africa and South America as well as manure and fertilizer nutrient specific histograms are shown in Figures S1, S2, S3. Corn yield from the crop responses functions was compared to IFPRI reported data in Figure S4; median yields are comparable but there is a much larger variability in the yields derived from the crop response functions. This reflects both the coarser resolution of the IFPRI data [23] and the more realistic representation of the frequency distribution of yields that are attained at individual locations across both continents from the FAO crop trial data.

**Figure 5:**
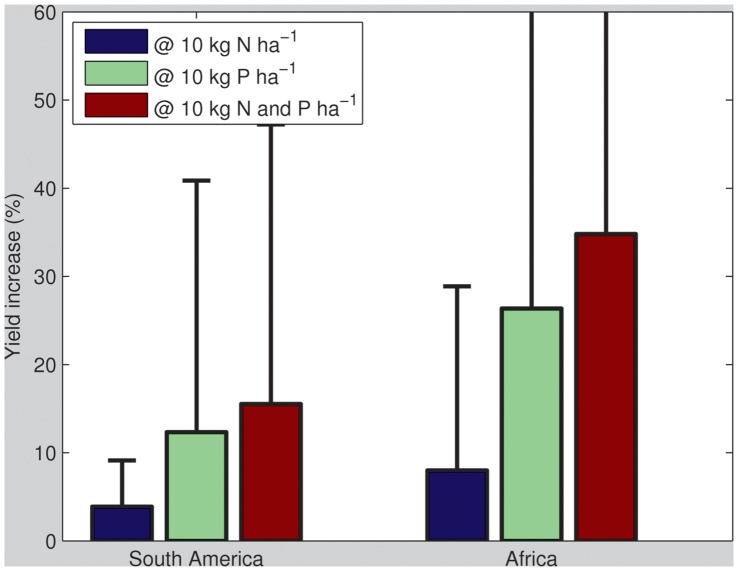
Mean corn yield increase (%) across trial sites at additional applications of 10 kg N ha^−1^, 10 kg P ha^−1^ or 10 kg N and P ha^−1^ (error bars refer to the standard deviation of the obtained yield increases observed across all trials).

To indicate the potential for production increase, we calculated the average percentage yield increases resulting from an additional application of 10 kg N ha^−1^, 10 kg P ha^−1^, and both. Adding only N will lead to increases in crop production by ∼4% and ∼8%. Adding only P, on the other hand, will lead to substantially larger increases of ∼12% and ∼26% for South America and Africa respectively (Figure 5). This highlights the critical importance of P, of which many subsistence farmers may not be aware. The addition of both nutrients leads to increases of ∼15% and ∼35%, respectively, indicating that the effect of both nutrients is additive once P is applied. Thus P is clearly the limiting nutrient in improving crop yields.

**Figure 6:**
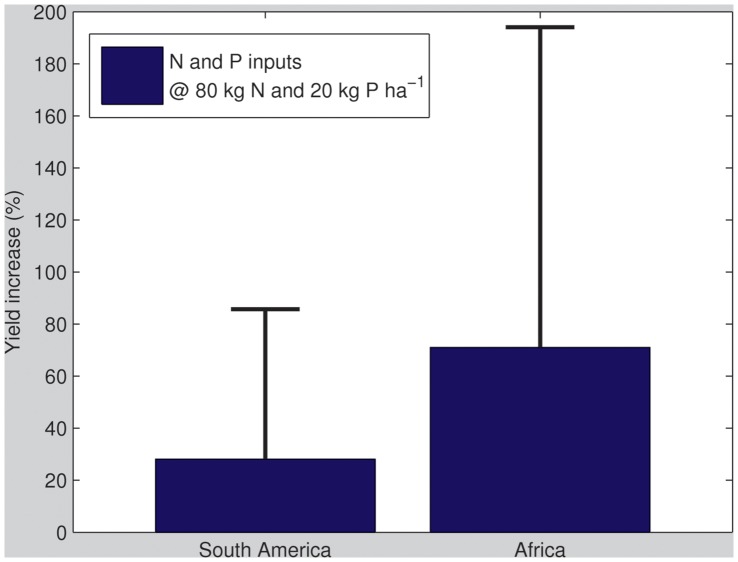
Mean corn yield increase (%) across trial sites at applications of 80 kg N ha^−1^ and 20 kg P ha^−1^ (error bars refer to the standard deviation of the obtained yield increases observed across all trials).

In Africa, adding 10 kg ha^−1^ of N or P will result in mean and median percentage increases of respectively ∼5.5 and ∼5.7% and ∼11.7 and ∼16.3%; this would thus bring a significant proportion of farmers with the lowest yields closer towards attaining average yield levels and effectively shift a bulk of smallholders out of current marginal productivity. Since these are indicative for rainfed yields, additional water resources to attain these yield increases would not be required [6]. A final experiment considers the percentage yield increase that would be obtained if 80 kg ha^−1^ of N and 20 kg ha^−1^ of P - a relatively conservative estimate of average rates in the USA - were applied (Figure 6). In South America this would lead to average yield increases of 30%, up to a maximum of 90% while in Africa these increases would be considerably larger, i.e. average yield increases of 70%, up to a maximum of 190%.

Even though these yield increases are considerable, they are lower than the yield potentials generally estimated in other studies. For example, yield gaps of 180 to 540% for maize have been estimated for sub-Saharan Africa [34] while a yield gap of 118% was found by Tittonell et al. [35] for western Kenya. Since most of the crop trials were done more than 20 years ago, our results will provide conservative yield estimates. In soils that have become increasingly depleted, and with new and better crop varieties that have become available since then, the response to fertilizer may be even stronger than predicted here.

### Implications

The implications for cropland expansion will be significant. Unless both N and P nutrient inputs are increased considerably and other complementary inputs and rural services such as seed, irrigation, market access and extension are available to improve crop yields, then necessary crop production gains will largely come from cropland expansion. This would have considerable negative impact on forest and grassland habitats and biodiversity [5]. In contrast, improving cereal yield by just 5% globally, over 33 million ha of forest or grassland would be saved. To put the benefits of these higher yields in context, our first scenario of applying an additional 10 kg N ha^−1^ and 10 kg P ha^−1^ (leading to a corn yield increase by 15% and 32% in South America and Africa) would save more than 4 million ha and 25 million ha of cropland conversion in South America and Africa respectively. Alternatively, if such yield improvement occurs in the currently cultivated cereal areas on these two continents, the improved productivity could feed an additional 64 million and 150 million people respectively.

### Costs

Such a scenario would require a total investment of US$148 million in sub-Sahara Africa (using an average of fertilizer prices paid by farmers in 2012 of Urea ($620/ton or 0.29$ kg^−1^ N) and DAP ($950/ton or 0.22$ kg^−1^ P) and US$79 million in South America (using an average fertilizer price paid by farmers in 2012 of Urea ($460/ton or 0.22$ kg^−1^ N) and DAP ($680/ton or 0.16$ kg^−1^ P)). The second scenario or larger nutrient inputs would amount to investments of US$798 million in sub-Sahara Africa and US$428 million in South America respectively. Maize yield increases range from 15% to over 70%, and such an investment would therefore bring considerable additional revenue to maize farmers. We acknowledge the fact that the prices of both fertilizer and maize vary with location so the actual profitability of fertilizer investment would vary spatially.

However, the direct investment in fertilizers is actually very small and is less than 1% of Agricultural GDP of both Sub-Saharan Africa and South America for the micro-dosing scenario. The calculation of the investment in terms of the percentage of Agricultural GDP that would be needed in SSA for scenario 1 equates to dividing 148 million USD by the Agricultural GDP of $150 billion USD multiplied by 100. The percentage of investment in terms of Agricultural GDP for the other scenario for SSA and the scenarios for SA were calculated in the same way (see Table 2).

**Table 2:** Cost of the proposed scenarios expressed as the percentage of Agricultural GDP.

Region	Cost as a % of Agricultural GDP	
	Scenario 1	Scenario 2
SSA	0.10%	0.53%
SA	0.04%	0.22%

## Discussion

In reality a full development blueprint would need to have a broader scope and costs would be compounded with investments in roads, agricultural extension, (local) market access, etc. [5], [6]. Nevertheless, external investment in this low technology solution has the potential to kick start development and could complement other interventions such as better crop varieties, improved economic instruments to support farmers as well as new technologies involving mobile phones, crowdsourcing and data mining of internet searches [36], [37]. To improve our understanding of how best to target, design and support rural development, it is insightful to compare the costs calculated here with the costs involved in a project such as the Millennium Villages (MV). The MV is an integrated approach to eradicate poverty by involving an entire community in improving their livelihoods and health in a sustainable way (http://www.millenniumvillages.org). The focused investments calculated here are significantly lower per person per year compared to the costs in the MV, which vary between 35 to 100 USD per person per year [38]. However, we clearly acknowledge that the MV has a much broader scope, engages entire communities and contributes to many other aspects of well-being and improved livelihoods as set out in the Millennium Development Goals, which would not be part of the scenarios suggested here.

We have clearly demonstrated the importance of phosphorus for closing yield gaps in Africa. The phosphorus deficiency reflects soil P supply problems that are of widespread concern in highly weathered tropical soils notorious for low levels of available P and exhibit a strong P fixation capacity [39]. For instance, approximately 82% of the land area of the American tropics is deficient in P in its natural state [40]. Combined with the fact that P reserves are likely to become exhausted during the next 30–300 years [41], this paints a bleak picture indeed. Nevertheless, if recycling programs were put in place for animal and human excreta some of these effects might be mitigated, e.g. [42]. Raising awareness of the need to provide a more balanced stoichiometry is also a critical element in improving yields. If farmers continue to add increased supplies of N without P, they will soon reach a saturation point in yields and effectively waste valuable resources as well as contaminating groundwater due to the leaching of nitrogen. This finding also has implications for the current fertilizer subsidy programs in many developing countries. Most of these programs focus mainly on N and do not emphasize the importance of P sufficiently. Our results demonstrate that better balanced subsidy schemes taking account of both N and P would have a larger effect on crop yields.

Climate change is expected to generally have a negative impact on corn in Africa with estimates of lowering yield ranging from 3 to over 12% [43]. Corn will be the most heavily impacted crop in sub-Saharan Africa, where yield losses would occur in 65% of corn growing regions for a 1°C warming, increasing to 100% losses in areas subject to drought stress [44]. Although these pressures are considerable, and will require adaptation and fundamental changes to agricultural management, our results indicate that significant increases in yield are possible by improved nutrient management; especially during growing seasons when soil water availability is not constraining crop yields. Thus, achieving trend-growth in crop productivity through sufficient and balanced nutrient applications coupled with effective storage policies could partly offset negative climate change impacts on food security in Africa. The risk averseness of poor farmers that are prone to drought is one of the reasons why these farmers will be hesitant to invest in higher fertilizer applications. However, we have shown that a relatively small external investment would yield large improvements in both crop production and sustainable use of soils.

In contrast to previous crop productivity assessments for Africa and South America, our study allows farm input management interventions to be directly based on small scale on-the-ground observations accounting for both site-specific conditions, as well as reflecting variability in soil conditions and climates. Furthermore, the study provides yield gaps that are realistically attainable as the assessment is based on farmers’ field trials and the costs associated with these interventions amount to less than 1% of Agricultural GDP in both Sub-Saharan Africa and South America. Crop field trials might be considered costly by some but provide essential and hard-won insights, and when analyzed comprehensively, have the potential - through better formulated policies and agreements - to reward global society with improved food security status for many.

## Supporting Information

Figure S1 **Current N and P inputs from chemical fertilizer (Nfer, Pfer) and manure (Nman, Pman) extracted and averaged from** [**22**] **for the 1358 trial locations.**
(TIF)

Figure S2 **Histograms of the N and P nutrient inputs from chemical fertilizer at the 1358 locations** [**22**] **.**
(TIF)

Figure S3 **Histograms of the N and P nutrient inputs from manure at the 1358 locations** [**22**] **.**
(TIF)

Figure S4 **Boxplots of regionally reported corn yields collected by IFPRI and corn yields obtained from the 1358 crop response functions (CRFs) with current N and P inputs from chemical fertilizer and manure (Nman, Pman) as reported by** [**22**] **.**
(TIF)
