# Supplementary figures and images for: Affordable Nutrient Solutions for Improved Food Security as Evidenced by Crop Trials
Source: PLoS One. 2013 Apr 2;8(4):e60075. doi: 10.1371/journal.pone.0060075 (PMC3615004; doi:10.1371/journal.pone.0060075)

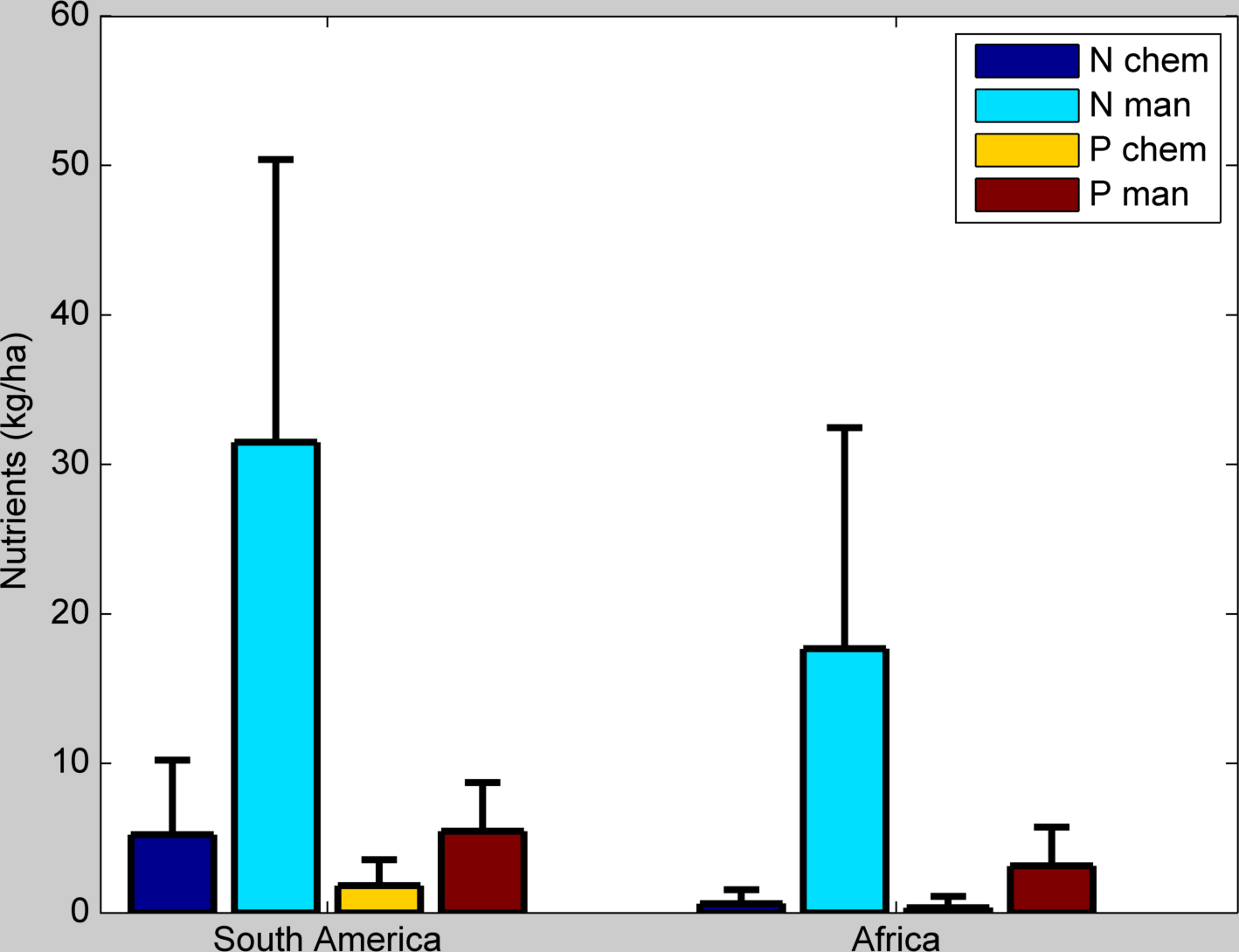

Supplement: Figure S1 — Current N and P inputs from chemical fertilizer (Nfer, Pfer) and manure (Nman, Pman) extracted and averaged from [22] for the 1358 trial locations. (TIF) [file pone.0060075.s001.tif]

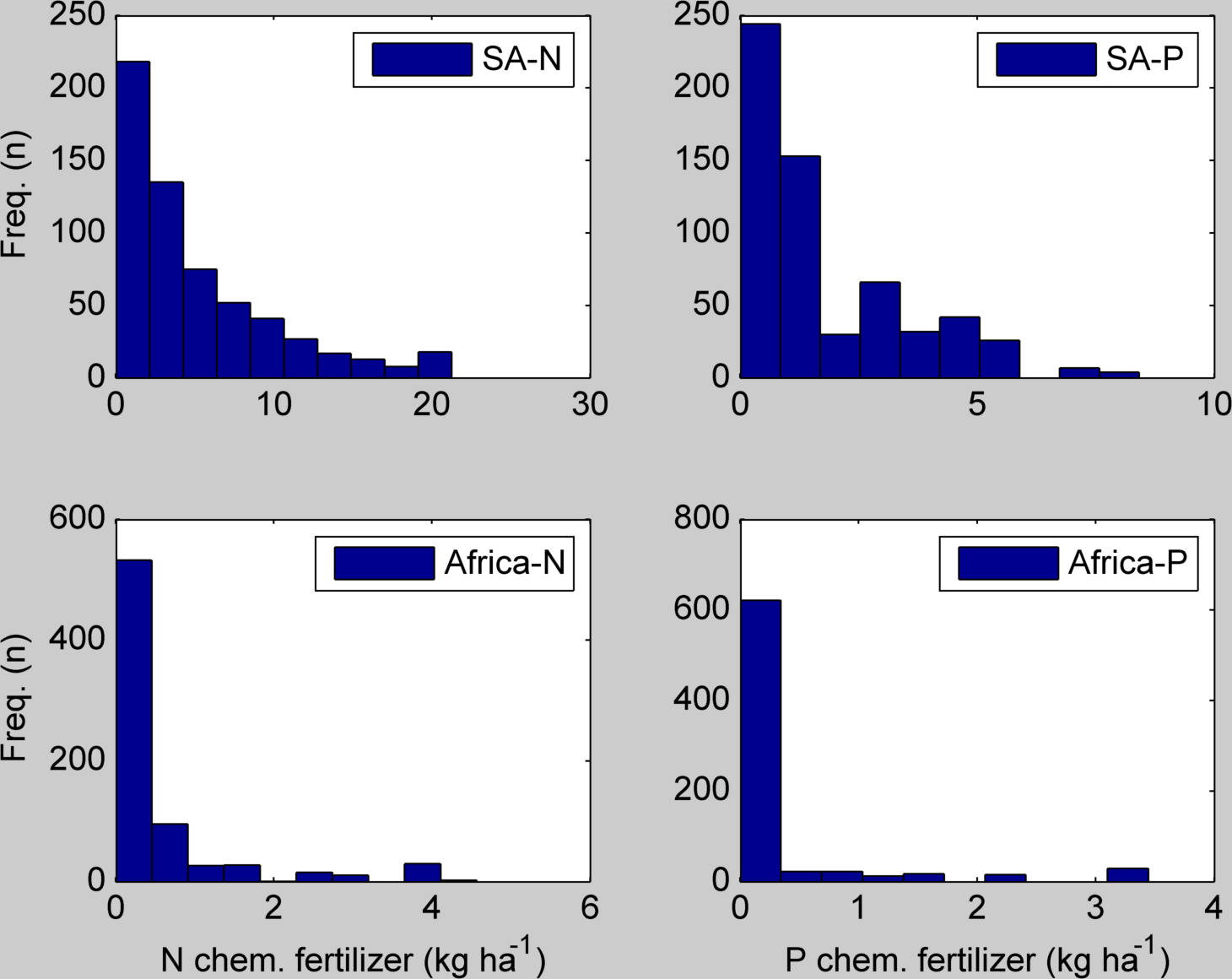

Supplement: Figure S2 — Histograms of the N and P nutrient inputs from chemical fertilizer at the 1358 locations [22] . (TIF) [file pone.0060075.s002.tif]

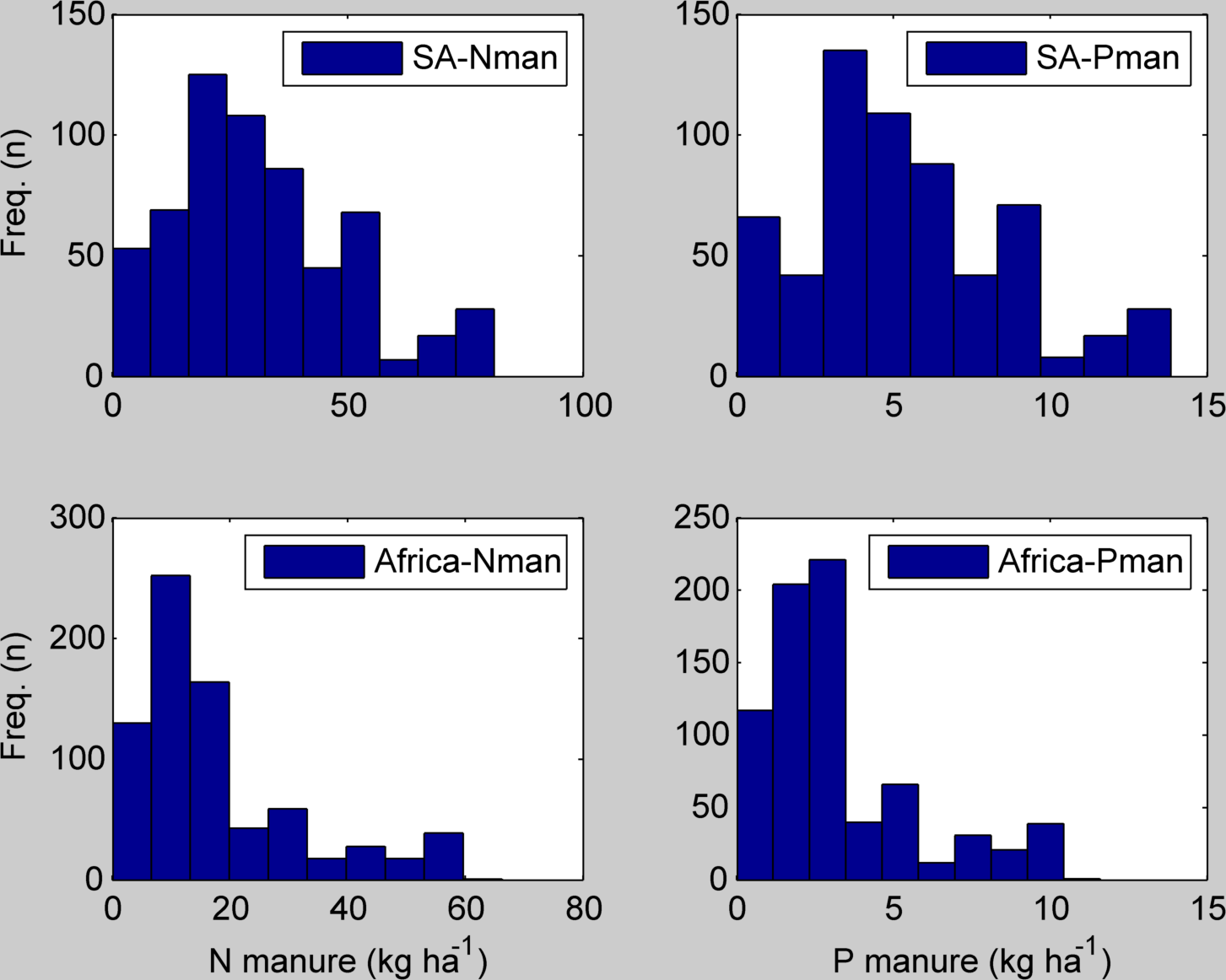

Supplement: Figure S3 — Histograms of the N and P nutrient inputs from manure at the 1358 locations [22] . (TIF) [file pone.0060075.s003.tif]

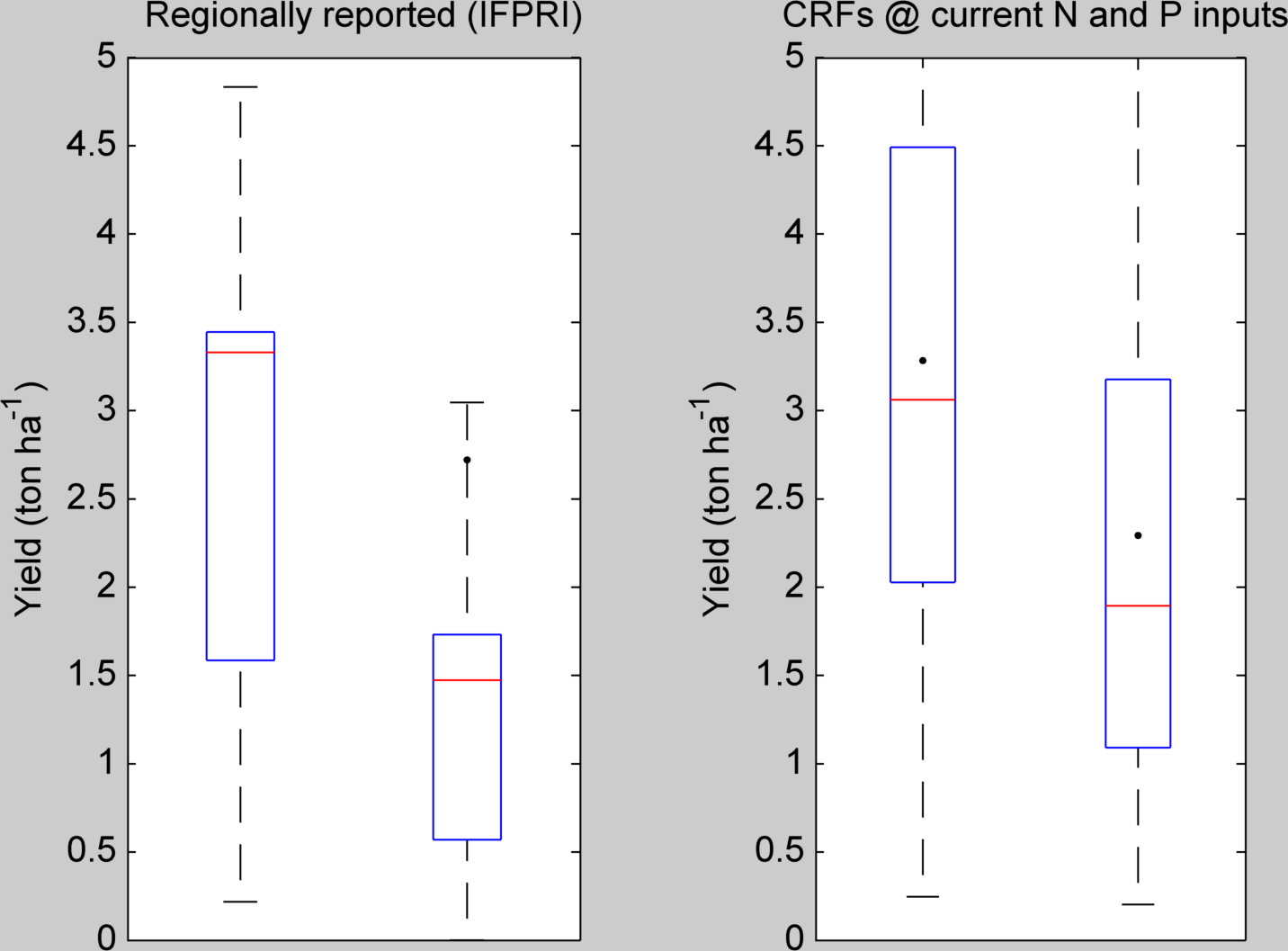

Supplement: Figure S4 — Boxplots of regionally reported corn yields collected by IFPRI and corn yields obtained from the 1358 crop response functions (CRFs) with current N and P inputs from chemical fertilizer and manure (Nman, Pman) as reported by [22] . (TIF) [file pone.0060075.s004.tif]
